# Techno-economic data and assumptions for open energy modelling of decarbonisation pathways in the Philippines

**DOI:** 10.1016/j.dib.2024.110459

**Published:** 2024-04-20

**Authors:** L. Dixon, R. Yeganyan, C. Cannone, M. Howells, V. Foster, F.A. Plazas-Niño

**Affiliations:** a Centre for Environmental Policy, Imperial College London, London, United Kingdom; b STEER Centre, Department of Geography, Loughborough University, Loughborough, United Kingdom

**Keywords:** Decarbonization pathways, Renewable energy targets, Net zero, Floating solar PV, Offshore Wind, Least-Cost Planning

## Abstract

This article introduces an openly accessible dataset aimed at supporting energy system modelling of decarbonisation pathways in the Philippines. The dataset was compiled through an extensive literature review, incorporating information from various sources such as the Philippines Department of Energy, academic publications, and international organisations. To ensure compatibility with OSeMOSYS modelling requirements, the data underwent processing and standardisation. It includes power plant data covering existing capacity from classified by grid, off-grid, and planned additions, as well as historical generation data. Additionally, the dataset provides historical and projected electricity demand from 2015 to 2050 segmented by sectors. It also offers technical potential estimates for fossil fuels and renewable energy sources, along with key techno-economic parameters for emerging technologies like floating solar PV, in-stream tidal, and offshore wind. The dataset is freely available on Zenodo, empowering researchers, policymakers, and private-sector actors to conduct independent energy modelling and analyses aligned with the U4RIA framework principles. Its open access encourages collaboration and facilitates informed decision-making to advance a sustainable energy future not only for the Philippines but also for broader global contexts.

Specifications Table

**Table:** 

Subject	Energy
Specific subject area	Energy System Modelling
Data format	Raw, Processed, Analysed
Type of data	Tables and Graphs
Data collection	Data was collected from websites, reports, and databases of international organisations and national entities, as well as from academic articles.
Data source location	Raw data sources are listed in the different sections of this article
Data accessibility	With this article and in a repository Repository name: Zenodo Data identification number: 10.5281/zenodo.10382708 Direct URL to data: https://zenodo.org/records/10382708

## 1 Value of the Data


 

• This dataset can be utilised to develop energy system models and assess decarbonization pathways for the Philippines. Depending on the design of the modelling process, other policy insights can also be obtained.
• The dataset included emerging technologies in the Philippines such as floating solar PV, instream tidal, and offshore wind which are absent from existing literature.
• Analysts, policymakers, and the scientific community can employ the dataset and the methods described for conducting energy studies not only in the Philippines but also in countries with similar characteristics. Furthermore, the findings of the energy system analysis can enable governments to strategically allocate financial resources for implementation, delineate the role of public funds, and improve access to global climate finance [1].

## 2 Background

Effective long-term energy planning hinges on accurate and accessible data to enable robust energy systems modelling. However, national-scale modelling efforts often face significant challenges due to limitations in data quality and accessibility [2]. This study addresses these challenges by offering an openly accessible dataset specifically designed for long-term energy planning in the Philippines. This data can be utilised by various stakeholders, including researchers, policymakers, and private-sector actors, by equipping them with the necessary tools for independent analysis and informed decision-making. By fostering transparency and collaboration through open access, we seek to stimulate more unified research efforts and enhance the field of energy modelling. Furthermore, this initiative aligns with the U4RIA framework of Ubuntu, Retrievability, Reusability, Repeatability, Reconstructability, Interoperability, Auditability [3], encouraging broader utilisation and advancement in energy modelling practices. This data-driven approach can also serve as a valuable template for future energy system modelling endeavours in developing nations. The use of localised data specific to the Philippines was prioritised, as opposed to generic international data, to ensure the dataset's direct relevance and applicability to local policy decisions. This dataset builds upon the existing Stater Data Kit for the Philippines created by [4], updating data values to 2023 and adding new technology options floating solar PV, tidal in-stream as well as fixed and floating offshore wind. This data set was constructed to feed into an OSeMOSYS model, so can be readily employed in this context or in similar long term energy modelling frameworks.

## 3 Data Description

This paper presents datasets that can be utilised for energy modelling of a long-term decarbonisation and clean energy transition planning in the Philippines using OSeMOSYS. However, it is important to note that the data provided in this document exist independently of the tool. To enhance accessibility, the dataset can be accessed on Zenodo repository through the following link: https://zenodo.org/records/10382708 [5]. The data is sourced from publicly accessible sources, such as the department of energy in the Philippines and pre-existing model databases.

### 3.1 Residual capacity

**Fig. 1:**
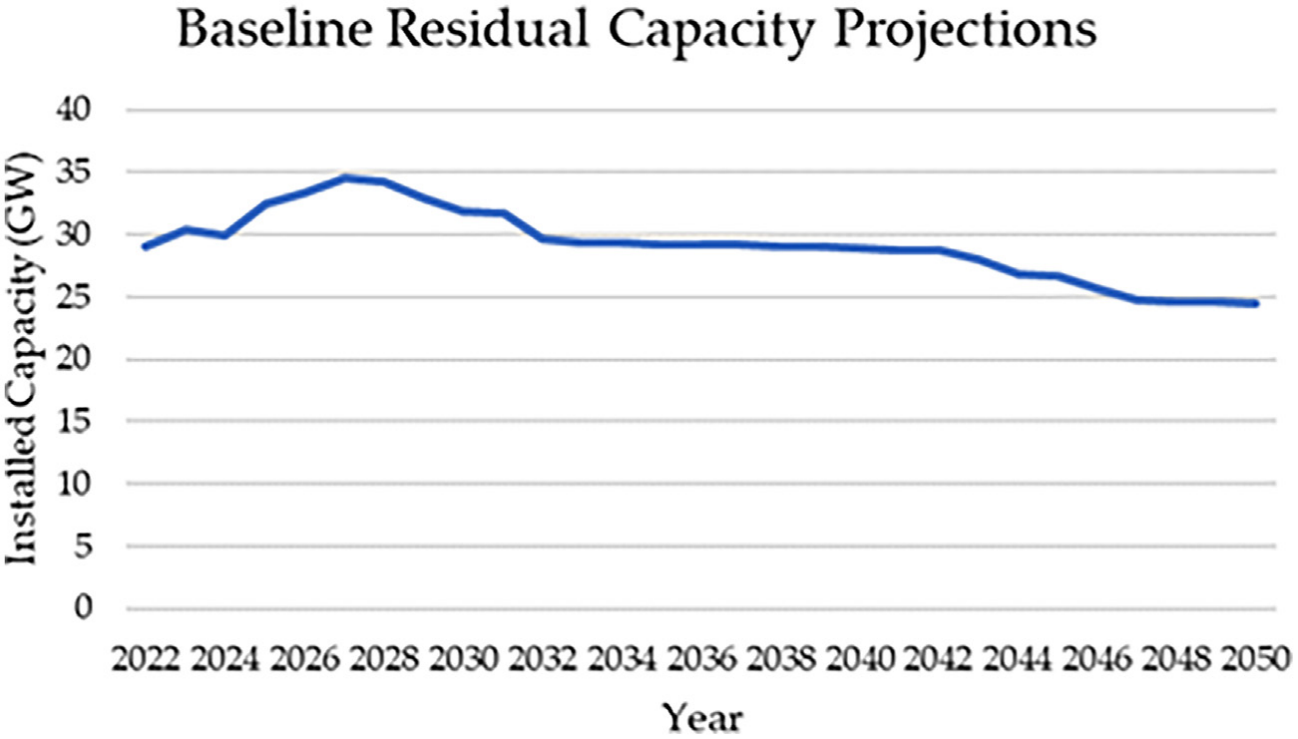
Baseline capacity installed in the OSeMOSYS model.

The residual capacity data represents the existing stock of power plants in the Philippines from 2015 to 2050, taking into account capacity connected to the Mindanao, Luzon, and Visayas grids as well as off grid and approved capacity projections by the Department of Energy [6], [7], [8], [9], [10]. Table 1 shows an excerpt of the installed capacity data for key years by Technology. Fig. 1 represents the baseline capacity installed in the OSeMOSYS model before optimization. The complete dataset is accessible in the Excel Sheet ‘Existing Power Plants’ in the repository.

**Table 1:** Installed Capacity in key years by technology [6], [7], [8], [9], [10].

Power Plant	2023	2030	2040	2050
Coal Power Plant	12.7726	14.7776	14.7776	14.1113
Natural Gas	4.2675	4.8794	2.8564	2.4125
Large Hydropower Plant	3.83527	3.42327	3.42327	3.42327
Medium Hydropower Plant	0.4694	0.42073	0.26543	0.16093
Small Hydropower	0.14144	0.12704	0.08794	0.07604
SCGT	2.93137	1.23747	1.08877	0.35277
CCGT	0.65	0	0	0
Geothermal	1.9658	1.3463	1.0308	0.1721
Onshore Wind	0.5369	0.7001	0.6671	0.2732
Biomass Power Plant	0.68528	0.68948	0.43928	0
Solar PV	2.02467	4.07528	4.07428	3.41458
Gas Turbine	0.13	0.13	0.13	0
<1kw LFO generator	0.357568	0.255609	0.152931	0.031128
Solar PV with storage	0.010139	0.010139	0.010139	0.000761
Off-grid Hydropower	0.026625	0.026625	0.025275	0

### 3.2 Generation

Generation data represents the actual generation in petajoules in historical years from the different power technologies represented in the model. The Data for 2022 for each plant type is summarised in Table 2, and can be found in the Excel sheet ‘Historical Elec. gen by Tech’ in the Zenodo repository.

**Table 2:** Generation in 2022 from each plant type [11].

Plant Type	Generation (PJ)
Biomass	4.76
Coal	239.15
Geothermal	37.53
CCGT	2.65
SCGT	22.41
Natural Gas	64.38
Solar PV	6.56
Large Hydropower(>100MW)	26.11
Medium Hydropower (10–100MW)	3.87
Small Hydropower	1.02
Offgrid Hydropower	0.53
Onshore Wind	3.71
Offgrid Oil-Based	1.58

### 3.3 Electricity demand

**Fig. 2:**
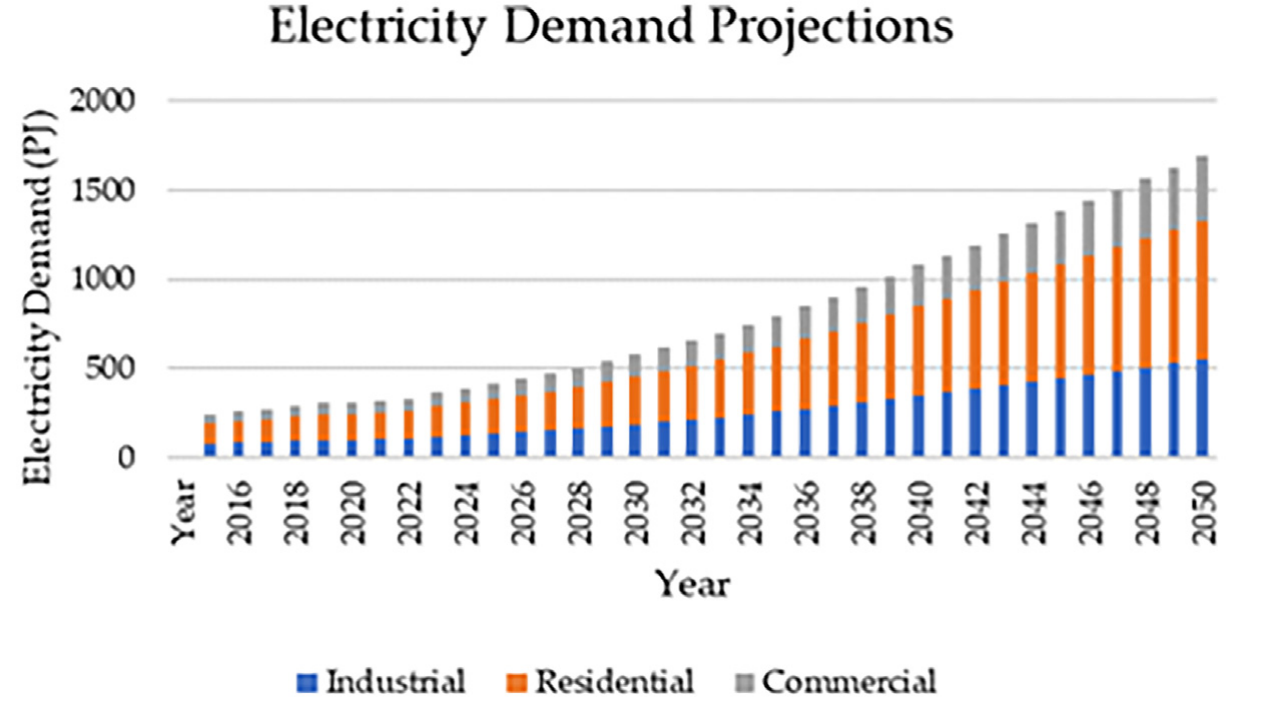
Projected Electricity Demand by sector 2015–2050 [12,13].

Electricity demand data represents both the historical and projected electricity demand for the period 2015–2050 from the industrial, residential, and commercial sectors. Table 3 presents an excerpt of the demand data by sector for key years. Fig. 2 presents the Projected electricity demand data used in the OSeMOSYS model. The complete dataset is accessible in the Excel Sheet ‘Elec. demand by sector’ in the Zenodo repository.

**Table 3:** Electricity demand projections by sector in key years [12,13].

	2023	2030	2040	2050
Industrial Demand (PJ)	118.7953	186.8193	350.4022	547.4801
Residential Demand (PJ)	168.2933	264.6606	496.4031	775.5967
Commercial Demand (PJ)	79.19687	124.5462	233.6014	364.9867

### 3.4 Fossil fuel potential

Data for the resource potential of fossil fuels represents the total energy (PJ) that can be generated from reserves in the country. This data was obtained from the EIA [14]. Biomass potential represents the maximum yearly energy (PJ) that can be generated from biomass in the Philippines. Summarised data is displayed in Table 4 below, and can be found in the Excel sheets ‘Fossil Fuel reserves’ in the Zenodo repository.

**Table 4:** Technical Potential for Coal, Crude oil, and Natural Gas [14].

Technology	Potential (PJ)
Coal	7620.156
Crude Oil	611.79
Natural Gas	3675

### 3.5 Renewable energy potential

Renewable energy potential data represents technical annual generation capacity potential in Gigawatts. The technical potential was chosen as it considers the limitations and feasibility of current technologies, infrastructure, and operational conditions. Biomass potential represents the maximum yearly energy (PJ) that can be generated from biomass in the Philippines. Summarised data can be found in Table 5, and can be located in the Excel sheet ‘RE Supply Potential’ and ‘Biomass Potential’ in the Zenodo repository.

**Table 5:** Technical Potential for Renewable Technologies [4,[15], [16], [17], [18], [19], [20], [21], [22].

Technology	Potential (GW)
Large Hydropower	14.6808
Medium Hydropower	1.89705
Small Hydropower	0.42002
Mini Hydropower (off-grid)	0.03731
Geothermal Power Plant	4.407
Onshore Wind	61.8
Fixed Offshore Wind	18
Floating Offshore Wind	160
Solar PV	337.2
Off-grid Solar with Storage	337.2
Floating Solar PV	83
Solar PV with storage	337.2
CSP	0
Tidal In-stream	50
**Technology**	**Potential (PJ)**
Biomass	136.7584

### 3.6 Key parameters for added technologies

Key technological parameters for the emerging technologies; fixed and floating offshore wind, floating solar PV, and instream tidal are summarised in Table 6. The capital cost represents the overnight cost for a technology from the year it becomes commercially available (in brackets) to the end of the modelling period. The fixed cost is the operation and maintenance cost independent of production output of the technology per year. The operational lifetime is the typical length of a power plant's operational life. Capacity factor refers to the ratio of actual electrical energy output over a given time period compared to the theoretical maximum electrical energy output in the same period. This data is available in the Excel sheets ‘Added Tech Add. Parameters’ and ‘Added Technologies Costs’ in the Zenodo repository.

**Table 6:** Key Parameters for added Technologies [19,[21], [22], [23], [24].

Technology	Capital Cost ($/kW)	Fixed Cost ($/kW)	Operational Life (Years)	Average Capacity Factor
Fixed Offshore Wind	2527 (2028)	63.4 (2028)	30	0.37
Floating Offshore Wind	3937 (2028)	30 (2028)	30	0.37
Floating Solar PV	864.3 (2023)	12.5 (2023)	30	0.165
In-Stream Tidal	2967 (2030)	62 (2030)	30	0.385

## 4 Experimental Design, Materials, and Methods

The dataset was compiled through a comprehensive literature review. Data was gathered from websites, reports, and other databases of international organisations and national entities, most prominently the Philippines Department of Energy, as well as from academic articles. The raw data was organised, analysed, processed, and standardised according to the requirements of the modelling. The following sections provide detailed information on the data sources, assumptions, and processing methods implemented in the construction of the dataset.

Residual capacity, historical generation, and historical demand data were updated using the most recent publications to improve the representation of the current Philippine energy system in an OSeMOSYS model, improving the application of model results to a real-world context. Moreover, updated estimates for energy demand projections and technology resource potential were integrated, as these parameters influence the build rate of new technologies in an OSeMOSYS model. Parameters for new technologies were defined to enable their inclusion in the least-cost optimization, thus enriching modelling outcomes with more comprehensive technology options for consideration.

### 4.1 Residual capacity

Future capacity projections for power plants in Mindanao, Luzon, and Visayas were determined by extrapolating current capacity indicated by the Department of Energy (DOE) using average operational lifetimes of each plant type and plant commission dates [6], [7], [8]. This projection also accounted for planned capacity expansions by the DOE and off-grid capacity [9,10]. However, the reported residual capacities for combined cycle gas turbine (CCGT) and large hydropower plant technologies were insufficient to match historical generation levels from 2015 to 2022 reported in the power statistics summary [11]. To rectify this, residual capacities were adjusted to 5GW and 10GW for CCGT and large hydropower respectively during those years in the model. This adjustment ensures consistency with historical generation without affecting future capacity projections, as residual capacities return to calculated levels from 2023 onwards. A limitation of this calculation method is the exclusion of power plants retired in the years 2018–2022.

### 4.2 Historical generation

Updated historical generation data was incorporated based on the latest gross generation statistics released by the Department of Energy in 2022 [11]. These data were converted from megawatt-hours (MWh) to petajoules (PJ) for model entry. Each plant type was assigned a maximum and minimum annual activity limit, with a 0.5 PJ difference above and below historical generation values. This adjustment aims to align the model with historical generation trends. The annual maximum capacity investments for the years 2015–2022 were set to 0 to prevent new capacity development in these years.

### 4.3 Electricity demand

Historical energy demand data from 2015 to 2022 was updated using the latest statistics from the Department of Energy's Power Statistics Summary in 2022 [12]. Projections for demand from 2023 to 2050 were based on growth forecasts from the Philippines Power Development Plan for 2020–2040 [13], linearly extrapolated up to 2050. This projection was segmented into commercial, industrial, and residential categories based on the sector demand breakdown in the DOE's key power sector energy statistics from 2021 [25].

### 4.4 Fossil fuel and biomass potential

Fossil fuel reserves are extracted from the US Energy Information Administration's data for the Philippines and are assumed to stay constant over the modelling period [14].

### 4.5 Renewable energy potential

Renewable energy potential data is mainly raw data sourced from various national institutions and databases, summarised in Table 7. The exception to this is hydropower where the raw potential for hydropower from the Department of Energy is split into potential for different-sized hydropower technologies in the model in the same ratio as residual capacity is split in 2022 (See data sheet ‘Existing Power Plants’ in the repository). The potential is assumed constant over the model period. Biomass potential is sourced from the Biomass Renewable Energy Alliance's estimates and is also assumed to stay consistent over the model period [15].

**Table 7:** Sources for renewable energy technology technical potential.

Technology	Sources
Large Hydropower	[16]
Medium Hydropower	[16]
Small Hydropower	[16]
Mini Hydropower (off-grid)	[16]
Geothermal Power Plant	[17]
Onshore Wind	[18]
Fixed Offshore Wind	[19]
Floating Offshore Wind	[19]
Solar PV	[20]
Off-grid Solar with Storage	[20]
Floating Solar PV	[21]
Solar PV with storage	[20]
CSP	[4]
Tidal In-stream	[22]
Biomass	[15]

### 4.6 Key parameters for added technologies

Costs, operational life and capacity factors for fixed and floating offshore wind are sourced from the Offshore Wind roadmap published by the World Bank and the Department of Energy, with the cost reduction calculated based on the average cost reduction in established markets [19]. Capital cost for floating solar PV is assumed to be 17.74% higher than ground-mounted PV, and fixed cost is the same, from the World Bank floating solar handbook [23]. Tidal instream costs and operational life are sourced from the Energy Transition Partnerships’ paper assessing marine renewables potential in the Philippines [22]. Floating solar capacity factor and operational life are taken from a USAID paper on floating solar deployment [21]. Capacity factor for tidal instream was sourced from a European Commission paper as a proxy [24].

## Limitations

Not applicable.

## Ethics Statement

Authors have read and followed the ethical requirements for publication in Data in Brief. This work does not involve studies with animals and humans.

## CRediT authorship contribution statement

**L. Dixon:** Conceptualization, Methodology, Formal analysis, Investigation, Data curation, Writing – original draft. **R. Yeganyan:** Project administration, Writing – review & editing. **C. Cannone:** Writing – review & editing. **M. Howells:** Supervision. **V. Foster:** Writing – review & editing. **F.A. Plazas-Niño:** Writing – review & editing.

## Data Availability

Techno-economic dataset for open modelling of decarbonization pathways in The Philippines (Original data) (Zenodo).
